# Classification and diagnostic prediction of breast cancer metastasis on clinical data using machine learning algorithms

**DOI:** 10.1038/s41598-023-27548-w

**Published:** 2023-01-10

**Authors:** Mahendran Botlagunta, Madhavi Devi Botlagunta, Madhu Bala Myneni, D. Lakshmi, Anand Nayyar, Jaithra Sai Gullapalli, Mohd Asif Shah

**Affiliations:** 1 VIT Bhopal University, School of Biosciences, Engineering and Technology, Kothrikalan, Madhya Pradesh India; 2 Institute of Aeronautical Engineering, Department of CSE, Hyderabad, Telangana India; 3 School of Computing Science and Engineering, VIT Bhopal University, Kothrikalan, Madhya Pradesh India; 4 Graduate School, Faculty of Information Technology, Duy Tan University, Da Nang, 550000 Viet Nam; 5 Oakridge International School, Gachibowli, Hyderabad, Telangana India; 6 Department of Economics, Bakhtar University, Kabul, 2496300 Afghanistan; 7 School of Business, Woxsen University, Kamkole, Sadasivpet, Hyderabad, 502345 Telangana India

**Keywords:** Cancer, Medical research, Oncology

## Abstract

Metastatic Breast Cancer (MBC) is one of the primary causes of cancer-related deaths in women. Despite several limitations, histopathological information about the malignancy is used for the classification of cancer. The objective of our study is to develop a non-invasive breast cancer classification system for the diagnosis of cancer metastases. The anaconda—Jupyter notebook is used to develop various python programming modules for text mining, data processing, and Machine Learning (ML) methods. Utilizing classification model cross-validation criteria, including accuracy, AUC, and ROC, the prediction performance of the ML models is assessed. Welch Unpaired t-test was used to ascertain the statistical significance of the datasets. Text mining framework from the Electronic Medical Records (EMR) made it easier to separate the blood profile data and identify MBC patients. Monocytes revealed a noticeable mean difference between MBC patients as compared to healthy individuals. The accuracy of ML models was dramatically improved by removing outliers from the blood profile data. A Decision Tree (DT) classifier displayed an accuracy of 83% with an AUC of 0.87. Next, we deployed DT classifiers using Flask to create a web application for robust diagnosis of MBC patients. Taken together, we conclude that ML models based on blood profile data may assist physicians in selecting intensive-care MBC patients to enhance the overall survival outcome.

## Introduction

Cancer is an uncontrolled growth of cells in the body that can rapidly spread to any organ and 90% of cancer patients die from metastasis^1,2^. Numerous types of cancer exist, but lung cancer, breast cancer (BC), and skin cancer are the most prevalent. According to World Health Organization (WHO) reports, the cancer death ratio is as high as 9.2 million for lung cancer, 1.7 million for skin cancer, and 627,000 for breast cancer^3,4^. Several image-guided deep learning models were developed for the prediction of cancer^5–8^. Along these lines, several machine-learning algorithms were utilized to distinguish benign from malignant cells based on histopathological reports^9^. The pathological report consists of clinical results, including microscopic observations and cancer stages. However, such information present in the documents is not properly documented in many of the hospitals. Complete patient information from the moment of diagnosis until the time of discharge is documented as both categorical and numeric data. Therefore, employing a text mining framework to extract meaningful information from medical records or text documents and apply it to machine-learning algorithms for cancer prediction is a challenging task^10,11^. Based on medical data, a stage-specific interpretation system was designed and this information serves as the primary resource for guiding patients' treatment methods^12,13^. Following confirmation of the disease's stage and subtype, the healthcare team initiates chemotherapy to limit the growth of cancer cells by modifying the expression of several genes. Text mining has helped to find biologically relevant alternative therapeutic candidates^14^ even though drug development remains a lengthy and expensive procedure. At present, Adriamycin, 5-fluorouracil, cisplatin, and taxanes (paclitaxel and docetaxel) agents are utilized to treat breast cancer^15^.

Medical Language Extraction and Encoding (MedLEE) and Clinical Text Analysis and Knowledge Extraction System (cTAKES, http://ctakes.apache.org/) were developed for the classification of breast cancer treatment studies at Columbia University and the Mayo Clinic, respectively. Nonetheless, cancer cells are resistant to a variety of therapeutic approaches. Cancer patients' platinum resistance is a major cause of clinical recurrence and death. Text mining, in conjunction with other bio-information techniques, assisted in the identification of platinum-resistance-related cancer genes^16^. The choice of therapy depends on the patient's physical characteristics and cancer stage. Throughout the course of treatment and follow-up, a Complete Blood Count (CBC) is taken to determine the response to therapy^17^.

Red blood cells, white blood cells, lymphocytes, monocytes, neutrophils, platelets, and other immune cells make up the blood cells. Understanding the dynamics of blood components facilitates the diagnosis of numerous disorders including cancer^12^. Blood cell counts and ratios are associated with poor overall survival (OS) in pancreatic and gastric cancers^17,18^. Platelet Distribution Width (PDW) is expected to function as a predictor of poor prognosis in breast cancer^19^ based on multivariate analysis. Patients with diffuse large B-cell lymphoma^20^, primary gastrointestinal diffuse large B-cell lymphoma, and urothelial and gastric cancer^21–23^ had their prognostic scores and ratios determined by absolute lymphocyte and monocyte counts and their ratios. It is known that lymphocyte count or percentage is a superior prognostic indicator for predicting the quality of life of advanced cancer patients^24^. In a retrospective study, the Receiver Operating Characteristic (ROC) analysis revealed that the monocyte counts and Monocyte Lymphocyte Ratio (MLR) can generate a moderate specificity of 71.68 percent, a sensitivity of 65.59 percent, and an Area Under the Curve (AUC) of 0.718 to differentiate cervical cancer patients from healthy individuals^25^. Calculating the performance of a machine learning model using ROC. Alternatively, blood counts and their ratios have been employed as a prognostic indicator for the diagnosis of COVID-19 individuals^26^. The relationship between haematological and spermatogenetic cells was discovered using machine learning algorithms^27^ to determine male fertility. Several machine-learning models have been utilized to detect and treat maternal anemia with Haemoglobin (Hb)^28^ and to diagnose haematological disorders^29^.

Similar to our method, a machine learning-based web tool was developed for predicting the kind of cancer using circulating miRNA in the blood and for distinguishing between the thalassemia trait and iron deficiency anemia^30,31^. Overall, it appears that blood counts, blood components (DNA, RNA, and Proteins), and/or their ratios, along with text mining algorithms, supplied a considerable piece of information for predicting cancer stage, recurrence, and overall survival. However, to the best of our knowledge, we were unable to locate any articles highlighting the application of text mining tools for the extraction of blood profile data and its usage in breast cancer classification.

Cancer is a complex disease to treat and manage such a complex disease proper diagnosis is needed. Usually, histopathological information about the malignancy of the lesion has been fundamental in cancer diagnosis all these years. Despite their utility, there are several limitations for it right from acquiring the tissue section to their storage, etc. Importantly cancer is an inherently heterogeneous disease. Analyzing a single site provides only a single spatial and temporal snapshot and is highly unlikely to reflect dynamic tumour heterogeneity. Hence, the development of reliable and robust non-invasive machine learning-based platforms is a vital step towards the promise of precision medicine. In light of this, blood profile data of MBC patients were processed through various ML algorithms, and compared the performance of each model using the fivefold cross-validation technique. The decision Tree (DT) classifier displayed accuracy of 83%. This is the first report conceptualizing histopathological data into a machine-learning model for the detection of breast cancer metastasis.

### Organization of paper

The paper is organized as in "Literature review” section highlights the literature review. In "Materials and methods” section elaborates on materials and methods. In "Results” section stresses on results, in "Discussion” section enlightens the discussion and in "Conclusion” section concludes the paper.

## Literature review

Text and data mining methods are becoming crucial in the healthcare system for the precise prediction of medical conditions. Text mining is a process that converts unstructured text data into meaningful and understandable information. There are numerous machine learning models, and their influence on predicting cancer therapy response is explored in^32^. Physicians and Scientists have several techniques for the identification of cancer, which includes Genetic analysis, symptom-based analysis, early-phase screening, etc. Table 1 provides a summary of each publication's key points.

**Table 1:** Metastatic and cancer prediction techniques.

Authors	Objective	Prediction model	Dataset	Prediction accuracy
Ahmad et al.^33^	To obtain the highest accuracy	CNN-LSTM, CNN-GRU and AlexNet GRU are used. Out of these three AlexNet GRU outperforms	Kaggle PCam imaging dataset	99.5%
Choudhury^34^	To diagnose and predict the cancer prognosis of Malignant Pleural Mesothelioma as early as possible (MPM)	8 different algorithms are used	Clinical data collected by Dicle University	79.29%
Bejnordi et al.^35^	To investigate the predictive power of deep learning algorithms Vs 11 members of pathologists in a simulated time-constraint environment	In a research challenge competition. 32 deep learning models have been submitted by the contestants out of which 7 models showed a greater performance	Detecting lymph node metastases: A CAMELYON16 dataset	Area Under the Curve (AUC) of 0.994
Abdollahiet al.^36^	To detect metastatic breast cancer using the whole-slide pathology images	Ensemble model consisting of VGG16, Resnet50, Google net, and Mobile net	CAMELYON16 dataset	98.84%
Papandrianos et al.^37^	To identify bone metastasis of prostate cancer	Convolutional Neural Network (CNN)	Nuclear Medicine Department of Diagnostic Medical Center, Larisa, Greece	97.38%
Gupta, and Gupta^38^	Deep learning approaches for predicting breast cancer survivability	Restricted Boltzmann Machine	The Surveillance, Epidemiology, and End Results (SEER) database	97%
Sharma and Mishra^39^	Performance analysis of machine learning based optimized feature selection approaches for breast cancer diagnosis	voting classifier	Wisconsin Breast Cancer (WDBC)	99.41%
Ak^40^	A comparative analysis of breast cancer detection and diagnosis using data visualization and machine learning applications	logistic regression model	Dr. William H. Walberg of the University of Wisconsin Hospital	98.1%
Maqsood et al.^41^	A breast cancer detection and classification towards computer-aided diagnosis using digital mammography in early stages	Transferable texture convolutional neural network (TTCNN)	DDSM, INbreast, and MIAS datasets	97.49%
Nanglia et al.^42^	An enhanced predictive heterogeneous ensemble model for breast cancer prediction	Heterogeneous Stacking Ensemble Model	Coimbra breast cancer dataset	78%
Feroz et al.^43^	Machine learning techniques for improved breast cancer detection and prognosis—a comparative analysis	K-Nearest Neighbor and Random Forest	Wisconsin	97.14%
Nasser^44^	Application of Machine Learning Models to the Detection of Breast Cancer	Random forest	Breast Cancer Database of Coimbra	83.3%
Seo et al.^45^	Scaling multi-instance support vector machine to breast cancer detection on the BreaKHis dataset	SVM	BreaKHis dataset	
Alfian et al.^46^	Predicting Breast Cancer from Risk Factors Using SVM and Extra-Trees-Based Feature Selection Method	SVM	Gynaecology Department of the University Hospital Centre of Coimbra (CHUC)	80.23%
Afolayan et al.^47^	Breast cancer detection using particle swarm optimization and decision tree machine learning technique	Particle swarm optimization and decision tree	Wisconsin breast cancer dataset	92.26%
Lakshmi, et al.^48^	Breast cancer detection using UCI machine learning repository dataset Wisconsin Diagnostic Breast Cancer (WDBC) is the cell nuclei features extracted from medical imaging	The paper discusses 11 different machine-learning algorithms for classification. The classification pipeline used is as follows: (1) Min–Max normalization, (2) dimensionality reduction PCA and t-SNE, and (3) the Randon Forest classification method	Wisconsin Diagnostic Breast Cancer (WDBC)	99% accuracy

Various imaging (X-ray, Bone, CT, MRI, PET scans) and lab tests are used to diagnose and increase the survival outcomes of breast cancer patients but not metastatic spread, a major cause of worse survival is to diagnose breast cancer metastasis. Hence, in this study, a machine learning-based web application is proposed for the early detection of breast cancer metastasis using blood profile data.

## Materials and methods

### Materials

#### The data acquisition system

**Figure 1:**
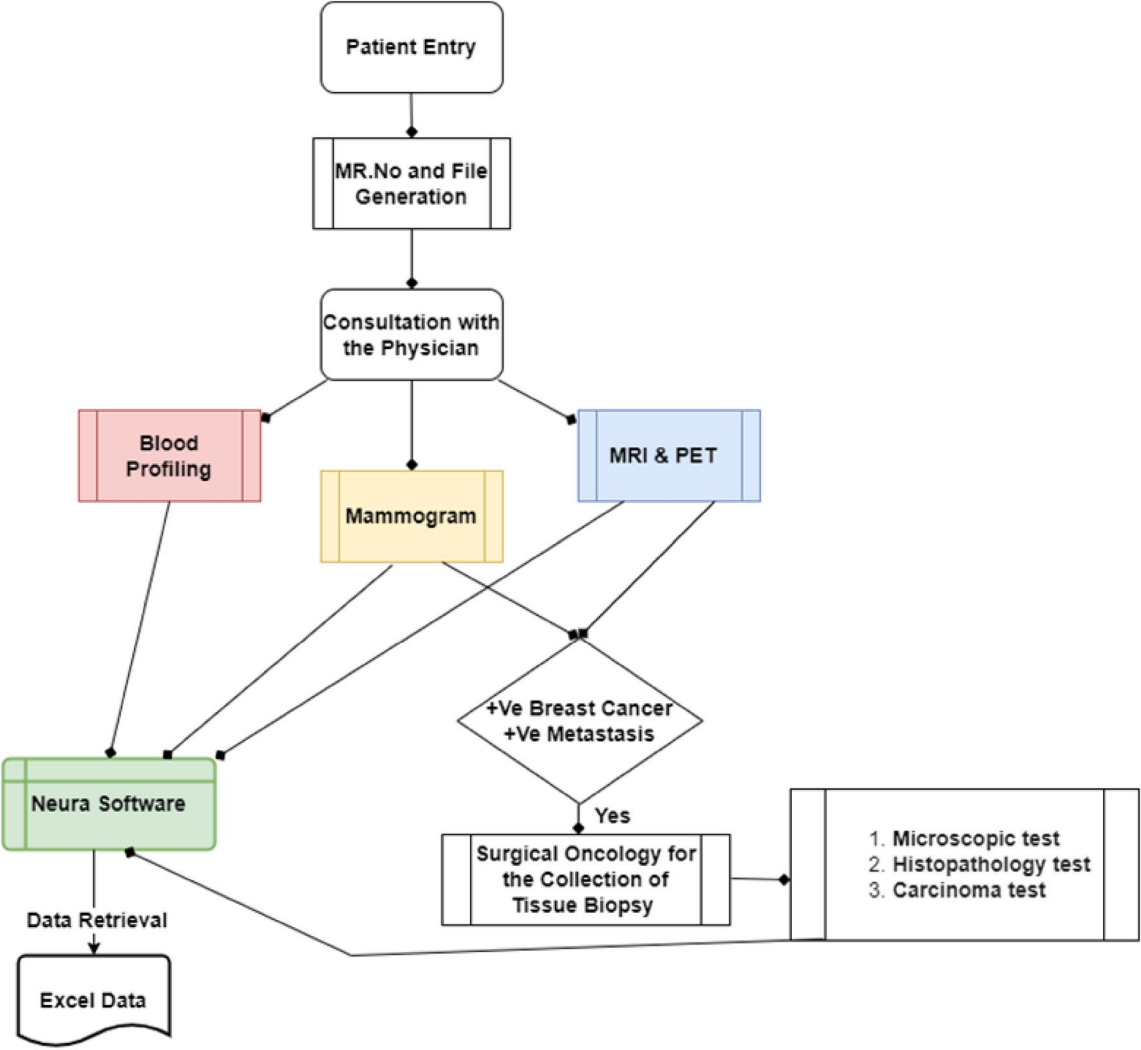
Data acquisition system.

Upon a visit to the hospital, the patient is requested to undergo a series of biochemical and anatomical observations. Biochemical tests such as complete blood picture (CBP) and serum proteins are commonly carried out to identify immune cell abnormalities and to monitor the functions of the kidney and liver. Simultaneously, both MRI and PET scans are also carried out to identify the presence of metastasis. Following observations, the physician recommends the patient undergo a biopsy examination under the supervision of a surgical oncologist. Subsequently, the biopsy test is sent for microscopic, histopathological examinations to determine the stage and grade of cancer. Complete information from the time of entry to exit is entered in NEURA software with a unique medical record number (MR) for the continuous follow-up of the patient's physiological status. A total of 26, 800 patient reports (year: 2012–2021) were received from the IT department of Basavatarakam Indo-American Cancer Hospital and Research Institute (BIACH & RI), Hyderabad, Telangana, India in an Excel format. The flow of the data acquisition system is shown in Fig. 1.

Haematological parameters such as ‘haemoglobin content’, ‘red cell’, ‘white blood cell’, ‘neutrophils’, ‘lymphocytes’, and ‘monocyte counts’ were extracted from the medical records for the selected cancer patients. On the other hand, blood profile data was extracted from 40 subjects with chemotherapy (Adriamycin, 100 mg/m^2^ for 4 cycles, followed by 4 cycles of paclitaxel (1000 mg/m^2^) to monitor the cycle-wise difference between haematological parameters.

### Methods

#### Data pre-processing and text mining

Data was acquired from BIACH & RI as a semi-structured Excel file containing a patient identifier (medical record number—MR.no) and a histopathology report (Hist_report). Data collected from the hospital is embedded in two columns: (1) Medical Record Number (MR No) and (2) Histopathological Report (Hist_report) with a total of 25,652 data entries in .csv format (Raw text). Data entries in this file not only constrain breast cancer but also other cancer types. Hence, split() function (tokenization) is employed to clean the undesirable data in Hist_report based on Breast and carcinoma keywords, then checked after splitting whether the string contains at least two parts or not. The dataset containing "breast" and "carcinoma" keywords in between the string is accepted, if not then that data is rejected. We employed enumerate and loop functions to iterate the raw text to obtain desirable data file and saved as a Breast.csv (Supplementary Table 1). By careful examination of the Hist_report in Breast.csv, embedded with several pathological observations such as clinical details, specimen details, microscopic observations, impressions, and gross findings. With the help of split and enumerate function we segregated Hist_report into Clinical, Specimen, Microscopic, Impression, and Gross Findings and tabulated (Supplementary Table 3).

#### Knowledge discovery and word cloud visualization

For knowledge discovery text data in Supplementary Table 3 is processed using various libraries (word_tokenize, WordNet, Lemmatizer, stopwords, punctuation, webtext, and FreqDist) present in Natural Language Toolkit (NLTK). We cleaned a text by removing stop words followed by lemmatizing. Code used for this process is [stop_words = set(stopwords.words('english') + list(punctuation) + custom_words)]. Stop words in English are (“a”, “the”, “is”, “an”, “so”, etc.), punctuation ('!"#$%&\'()* + , − ./:; <  =  > ?@, etc.) and custom words include ("suggestive", "analyze", "Lsd Exception", etc.). We used wold cloud to visualize the most often words used by clinicians and surgical oncologist to diagnose the cancer.

#### Machine learning algorithms for the classification of breast cancer

This comparison study included 9 classification methods, including Logistic Regression, KNN, Decision Trees, Random Forest, SVM (SVM linear, SVM radial), Gradient Boosting, and XGBOOST. The pseudocode for all the mentioned algorithms is shown Table 2.

**Table 2:**
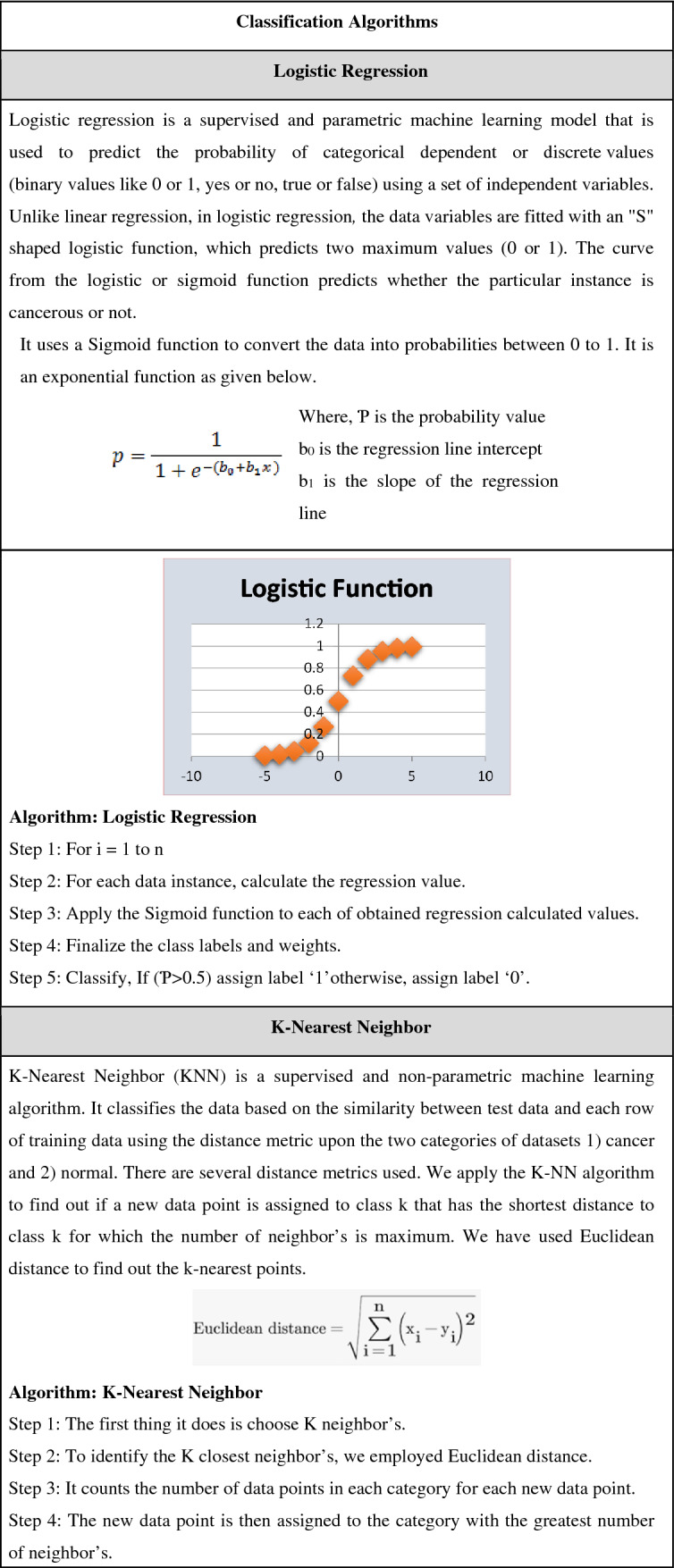

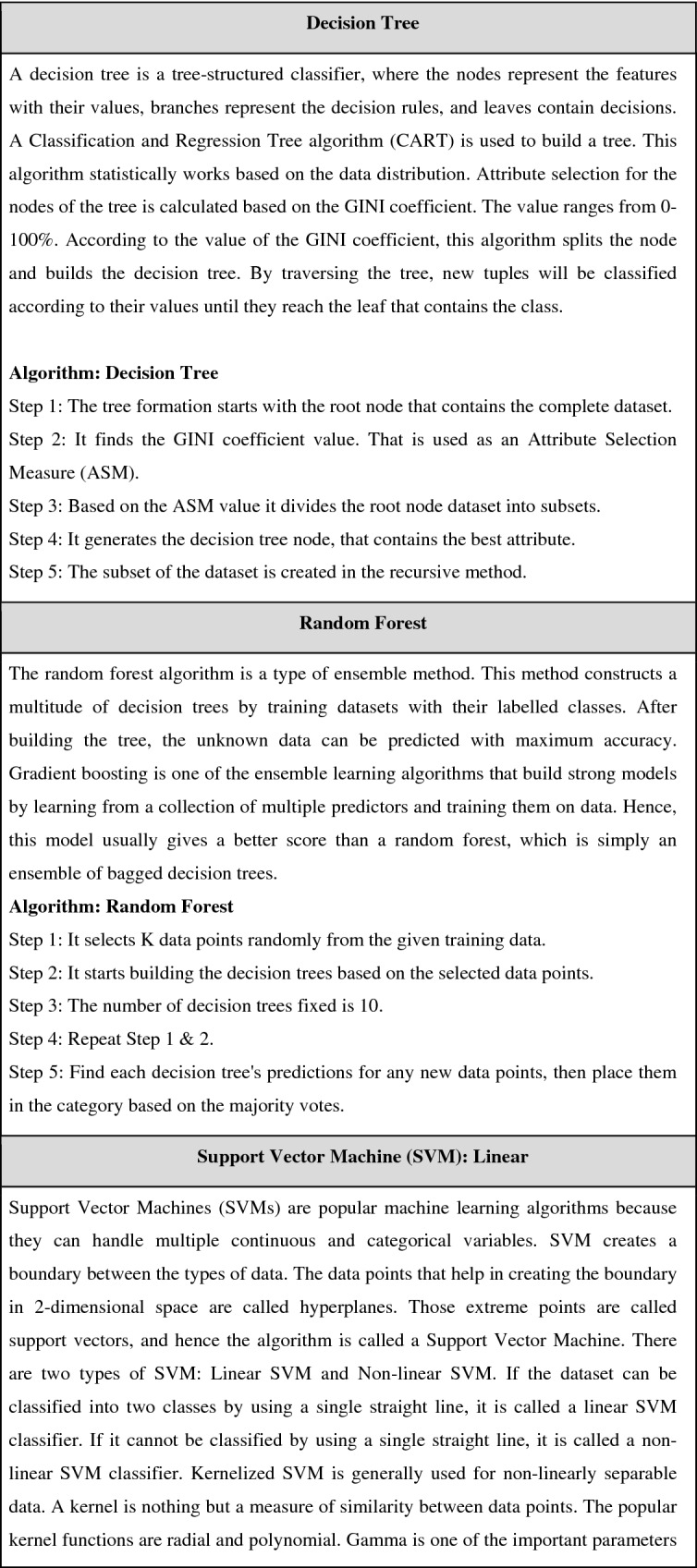

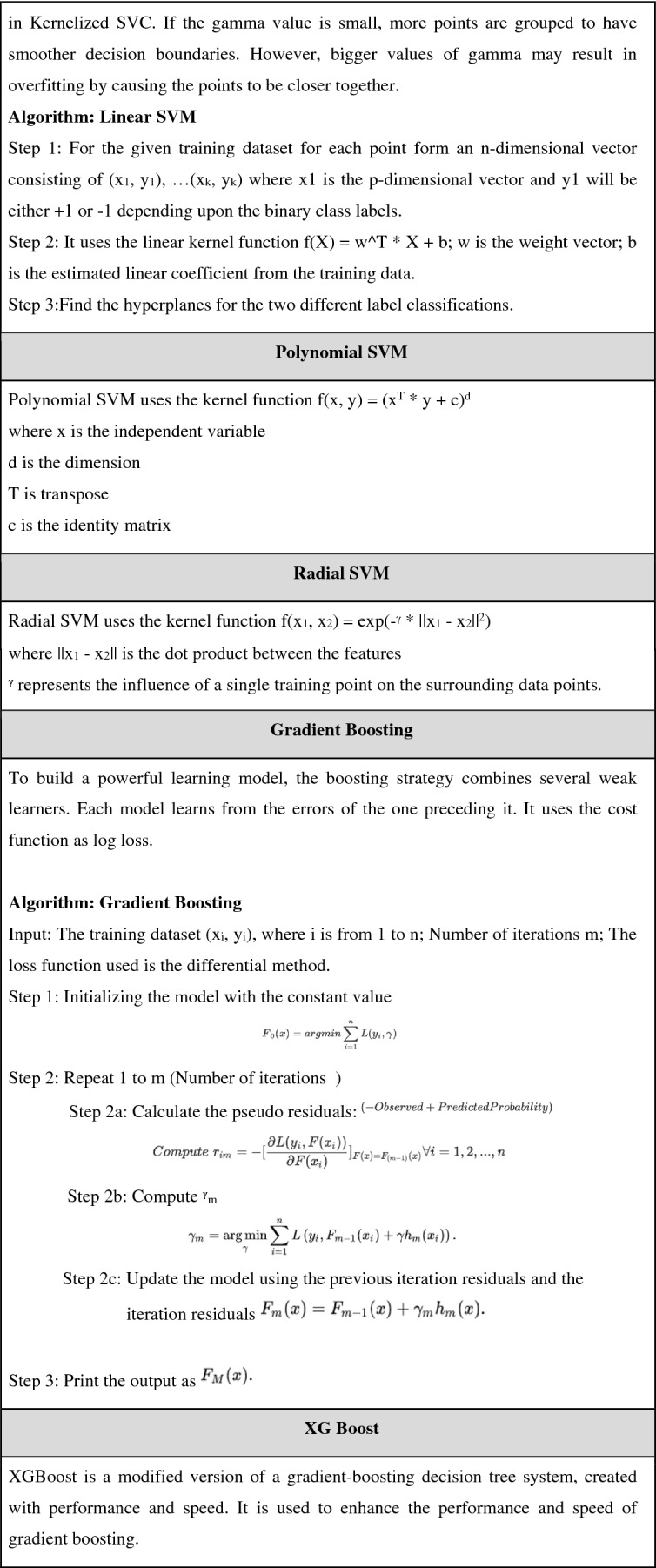
Pseudocode for machine learning models.

#### Model validation metrics

The performance of every model is evaluated by measuring the accuracy, recall (also known as sensitivity), precision, F1 score (harmonic mean of recall and precision), and AUC-ROC curve. These measures are calculated using the following formulae: Accuracy = (TC + TN) / (TC + TN + FC + FN), recall = TC / (TC + FN), precision = TC / (TC + FC) and f-measure = 2 * ((precision * recall)/ (precision + recall)). Where TC is the correct classification of breast cancer metastasis, FC is an incorrect classification of breast cancer metastasis, TN is the correct classification of normal subjects, and FN is an incorrect classification of normal subjects.

#### Statistical analysis

Python 3 Kernel in the Anaconda-Jupyter notebook is used for data analysis and scientific computing. Pandas, NumPy, and Matplotlib modules were imported for our data analysis. The Pandas module is used to read the data from excel. The data is pre-processed by dropping unnecessary columns. The overall distribution of haemoglobin content (Hb), TWBC, lymphocytes, neutrophils, and monocytes are calculated. An overall mean difference and Standard Deviation (SD) are calculated between normal and cancer subjects. Comparative cycle-wise mean value distribution for haematological parameters is studied to validate the impact of chemotherapy on these parameters.

## Results

### Pre-processing the clinical text data

**Figure 2:**
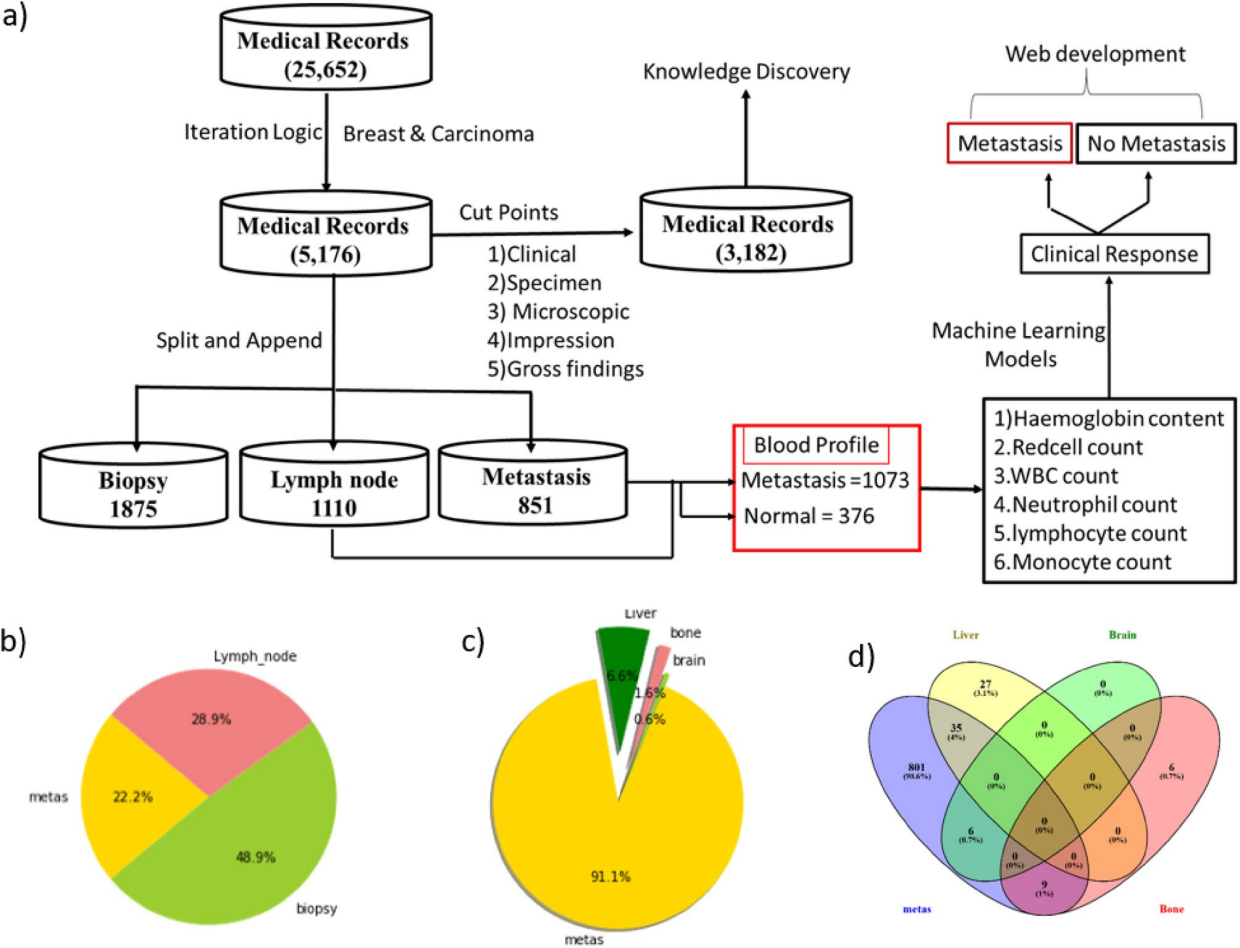
Text analytics based on clinical notes of breast cancer patients (**a**) A text mining framework for detecting cancer using blood profile data. (**b**) A pie chart depicting the proportion of patients who have metastasis and lymph node involvement. (**c**) A pie chart depicting the percentage of disseminated cancer cells in the liver, bone, and brain of patients with metastatic breast cancer. (**d**) Venn diagram representing the number of medical records intersecting between "metas" and bone, brain, and liver.

Pathological information collected from the hospital is embedded in two columns: (1) Medical Record Number (MR No) and (2) Histopathological Report (HR) with a total of 25,652 data entries. Following tokenization dataset is reduced to 5176 entries and saved as a Breast.csv (Supplementary Table 1). This file is used to identify the medical records matching the basic knowledge elements associated with cancer malignancy such as biopsy, lymph node, and metastasis. Among the 5176, 1875 were matched with biopsy, 1110 with lymph nodes, and 851 with metastasis (Supplementary Table 2). A flow diagram depicting a step-wise text mining and machine learning framework for the retrieval of blood profile data against breast cancer metastasis is depicted in Fig. 2a. A pie chart was used to represent the percentage of medical records that matched against biopsy, lymph node, and metastasis (Fig. 2b). Dissemination of cells from the site of origin is called metastasis. To identify the number of patient medical records with metastasis into visceral organs, we employed the “split” and “append” functions in the python terminal. Out of 851, 62 MR Nos. matched with liver, 15 MR Nos. with bone, and 6 MR Nos. matched with the brain (Fig. 2c) while the remaining 789 MR Nos. matched with Lymph node involvement. To identify patients with multiple Mets, we compared the dataset using the Venn diagram. The Venn diagram indicates 35 medical records intersecting between "metas" and Liver, 9 medical records intersecting between "metas" and Bone, 6 medical records intersecting between "metas" and Brain, and 27 records exclusively found in the Liver (Fig. 2d). Taken together, it is clear that the liver is the primary hotspot for the dissemination of cancer cells from the breast, and these cells may disseminate via the hematopoietic or lymphatic system.

### Knowledge discovery

**Figure 3:**
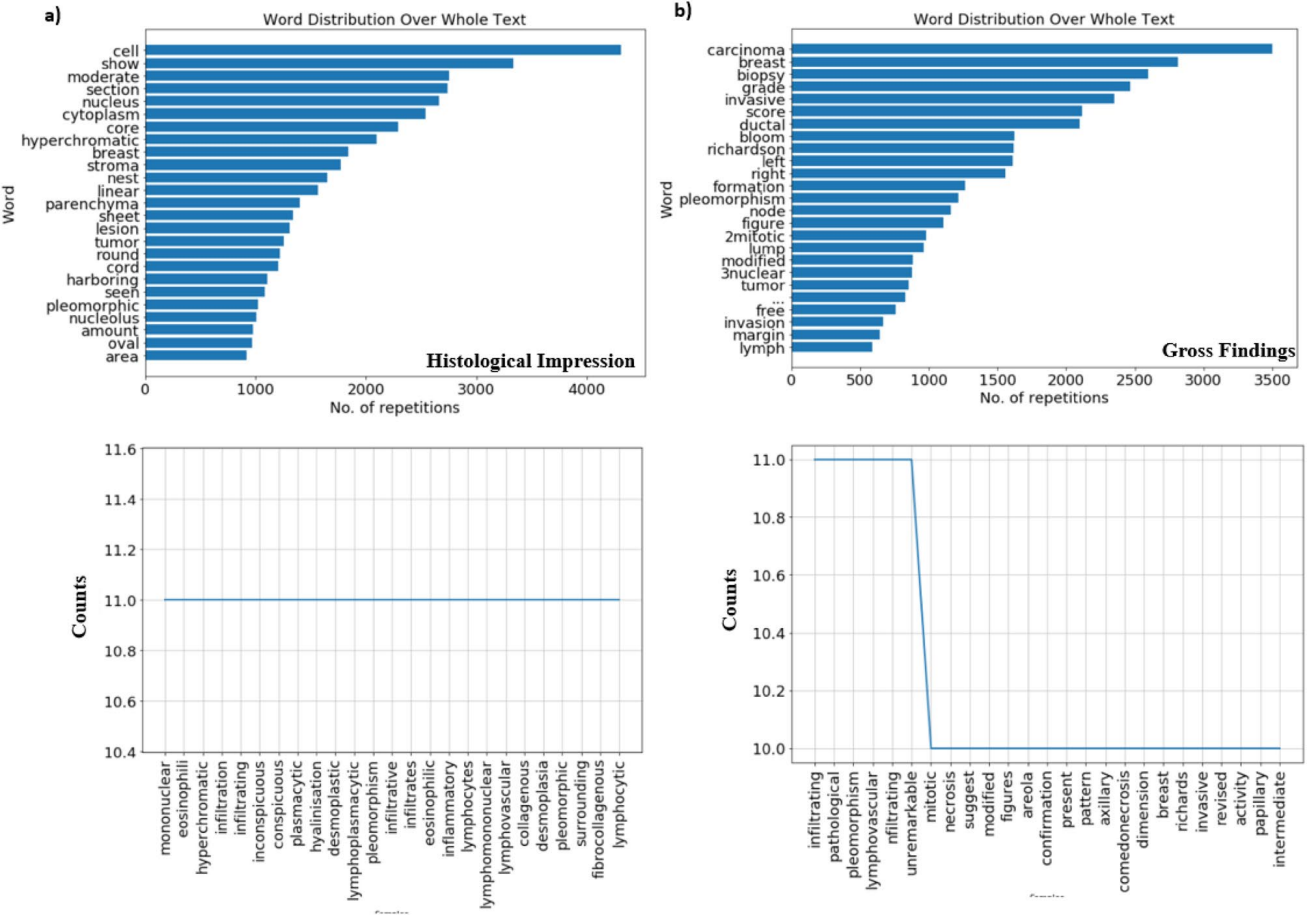
Text Mining and Knowledge Discovery of clinical data (**a** and **b**) the upper panel describes word distribution over the histological impression and gross findings. The bottom panel represents words with a frequency count above 10 and a length of more than 5 letters.

The impression and gross findings categories in supplementary Table 3 is used as input to find out word distribution over the whole text and the word frequency above 10 with a word length greater than 5 letters. Word cloud and word frequency provided great inferences. A word cloud showed the most popular words used by a pathologist under the impression category were nucleus, cytoplasm, hyperchromatic, and stroma (Fig. 3a). Based on the word count and frequency, we suggest that the Nuclear to Cytoplasmic (NC) ratio, hyperchromatic and pleomorphic nuclei are the first and most important parameters used for the identification of tumor cells in tissue biopsy. The cytoplasm is often found to be vacuolated and eosinophilic (the appearance of cell kinds of structures in the cytoplasm). Nucleoli are found to be inconspicuous. Infiltrated lymphocytes, plasma cells, a few eosinophils, and neutrophils are often found in the stroma. Based on clinical and microscopic observations, the pathologist discloses the overall grade and type of cancer (Gross Findings). The most popular words used by a pathologist in the gross findings are Bloom Richardson score (BRS) and grade (Fig. 3b). BRS is often used to grade tumors and types of cancer. It mainly depends on three variables, such as tubule formation (1 means > 75% tumor, 2 means 10—75% tumor, and 3 means < 10% tumor) nuclear pleomorphism (variation in size and shape of nuclei: 1 means minimal or mild, 2 means moderate, 3 means marked) and mitotic content (number of dividing cells). Taken together, our results suggest that the microscopic observation of cells in the tissue microenvironment is a very important indicator for the determination of tumor grade by pathologists.

### The overall distribution of haematological parameters in breast cancer patients

**Figure 4:**
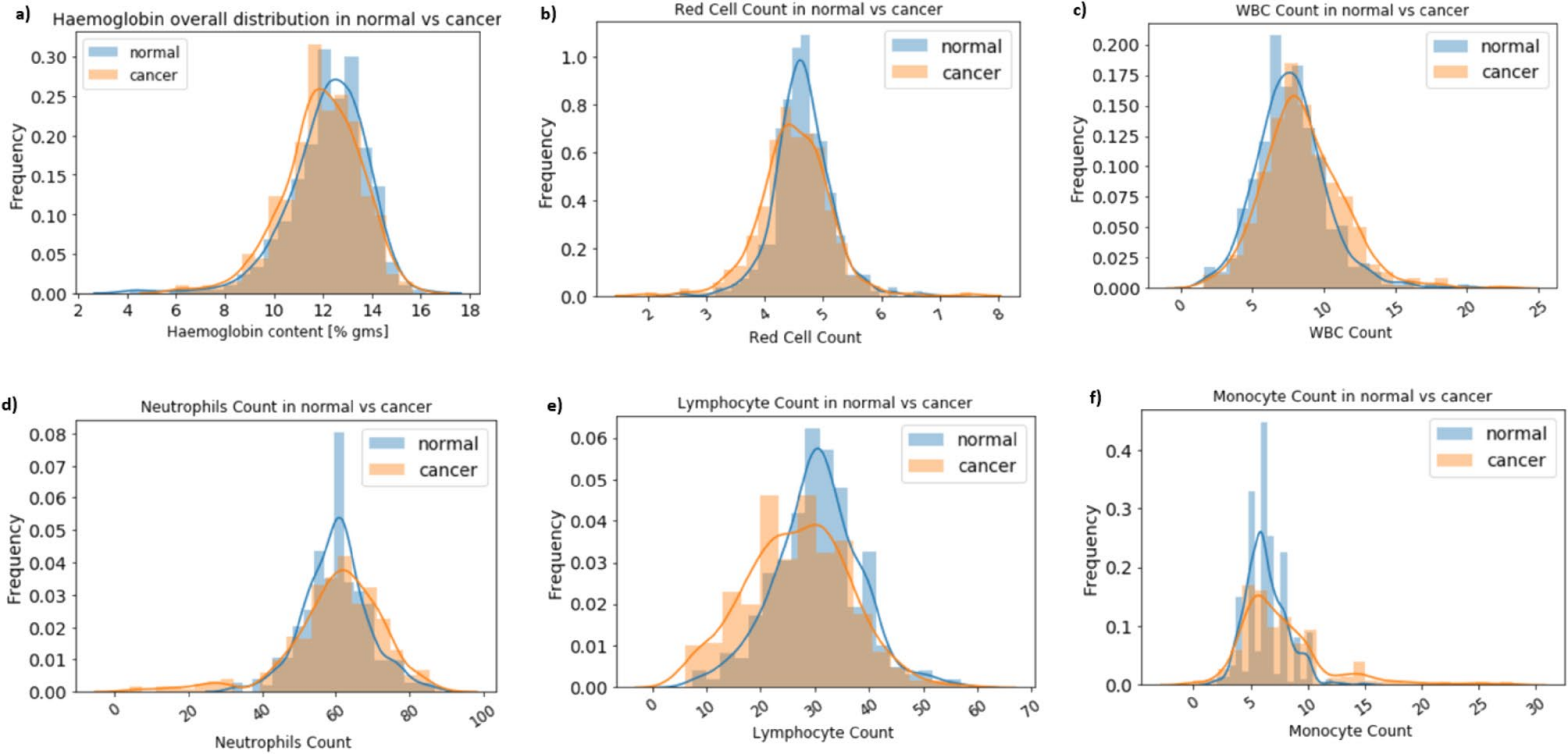
Overall distribution of haematological parameters between normal and cancer subjects. Histogram showing (**a**) haemoglobin content, (**b**) red cell count, (**c**) white blood cells, (**d**) neutrophils, (**e**) lymphocytes, and (**f**) monocyte counts. Here, frequency on the Y-axis represents the number of times a particular value occurred between normal and cancer subjects (no. of times repeated/total no. of. observations).

Blood is the main carrier for tumor cell dissemination and cancer metastasis. Based on text mining data (Supplementary Table 2) we extracted blood profile data from metastatic breast cancer patients. Blood profile data consist of Haemoglobin content, Red cells, White Blood cells, Neutrophils, Lymphocyte, and Monocyte counts. Moreover, blood profile data from normal female subjects were retrieved as a part of regular check-ups and tabulated in a single excel sheet. An equal number (376 + 376) of cancer and normal subjects were compared to identify the overall distribution of haematological parameters (Supplementary Table 4). Results showed that haemoglobin content, WBC, Lymphocyte, and Monocyte showed differential distribution between normal and cancer patients. Low Hb content, high WBC, Lymphocyte, and Monocyte are observed in cancer patients as compared to control (Fig. 4). Among all, red cell and monocyte count is increased and show a clear distinguishing pattern in cancer patients. The overall mean difference for Hb content in a cancer patient is 11.95 (12.19) as compared to normal with a p-value less than 0.05 (0.038). Similarly, for RBC 4.511 (4.643) with *p* value of 0.0007, for WBC 8.554 (7.724) with p-value of 0.0001, for Neutrophils 60.263 (60.194) with *p* value of 0.93, for Lymphocyte 26.75(30.42) with *p* value of 0.0 and for Monocyte 7.76 (6.36) with p-value of 0.0. Except for Neutrophils *p* value for all the Haematological parameters is significant. Overall, it suggests that Lymphocytes and monocytes are showing a distinguishing pattern between normal and cancer subjects. Overall it suggest that blood profile data can be used to distinguish breast cancer subjects as compared to normal subjects.

### Comparative cycle-wise mean value distribution for haematological parameters

**Figure 5:**
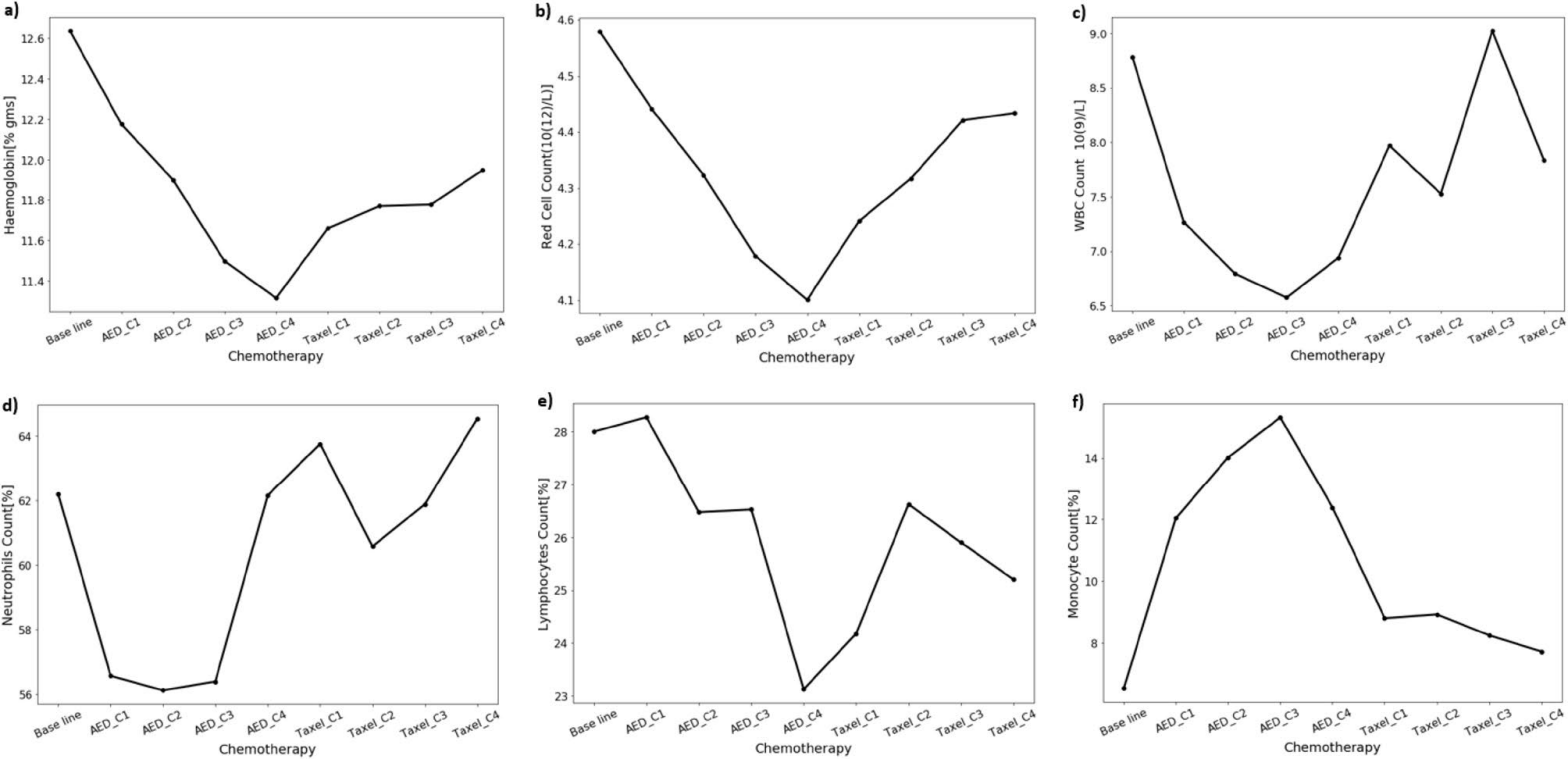
Comparative cycle-wise mean value distribution for haematological parameters (**a**) haemoglobin concentration. (**b**) The total number of red blood cells. WBC count (**c**), neutrophil count (**d**), lymphocyte count (**e**), and monocyte count (**f**). AED represents a combination of Adriamycin [100 mg] and cyclophosphamide [1000 mg] treatment.

Next, we wanted to study whether the selected parameters were responsive to chemotherapy or not. We collected the blood profile data before and after the therapeutic regime. The Chemotherapy protocol for breast cancer includes 8 cycles, and every cycle is repeated after a 3 weeks gap. Chemotherapy with AC (Adriamycin [100 mg] + cyclophosphamide [1000 mg]) is followed by Taxol (300 mg). Changes in haematological parameters such as haemoglobin, red cell, WBC, neutrophils, lymphocyte, and monocyte counts were measured at the end of every cycle (Supplementary Table 5). Cycle-wise mean value distribution across the haematological parameters is processed (Fig. 5). As shown in the figure, by the end of 4 cycles of AC treatment, a stepwise decrease in Hb content, RBC, and lymphocyte counts were observed, then slowly increased by shifting to Taxol treatment. Unlike Hb and RBC counts, lymphocyte and neutrophil counts were reduced by the end of the 1^st^, 2^nd^, and 3^rd^ cycles of AC treatment and then increased to close to the baseline by the end of AC and Taxol therapy. Most interestingly, the monocytes count increased above the baseline until 3 cycles of AC treatment, eventually decreased, and almost reached the baseline by the end of Taxol treatment.

Overall it suggest that the haematological parameters are responsive to the chemotherapeutic regime in breast cancer patients. To identify the therapy responsive feature, we performed a correlation analysis between baseline data (blood profile data at the time of visit to the hospital) vs therapeutic data (blood profile data in response to chemotherapy). Correlation analysis showed that lymphocyte, neutrophil, and monocyte counts are responsive to chemotherapy (Table 3). The Neutrophil and Monocyte counts showed a statistically significant difference between AC and Taxol treatment with a *p* value less than 0.05. However, haemoglobin concentration, the total number of red blood cells, and WBC count are not correlated in response to chemotherapy (*p* value > 0.05). Hence in our study, we used baseline data for the classification of breast cancer.

**Table 3:** Comparative mean-wise haematological parameters between AC (Adriamycin + cyclophosphamide) and Taxol-treated breast cancer patients. Values in the bold box represent a statistically significant *p* value.

Haematological parameter	Mean		Std	*p* value
	AC	Taxol		
Hb	11.9	11.95	1.14	0.6569
RBC count	4.32	4.39	0.46	0.099
WBC count	7.26	8.22	4.5	**0.013**
Neutrophils	58.69	62.58	9.11	**0.0002**
Lymphocyte	26.48	25.98	7.25	0.5215
Monocyte	12.06	8.04	2.7	**0.000**

### Classification of breast cancer by a machine learning model

**Figure 6:**
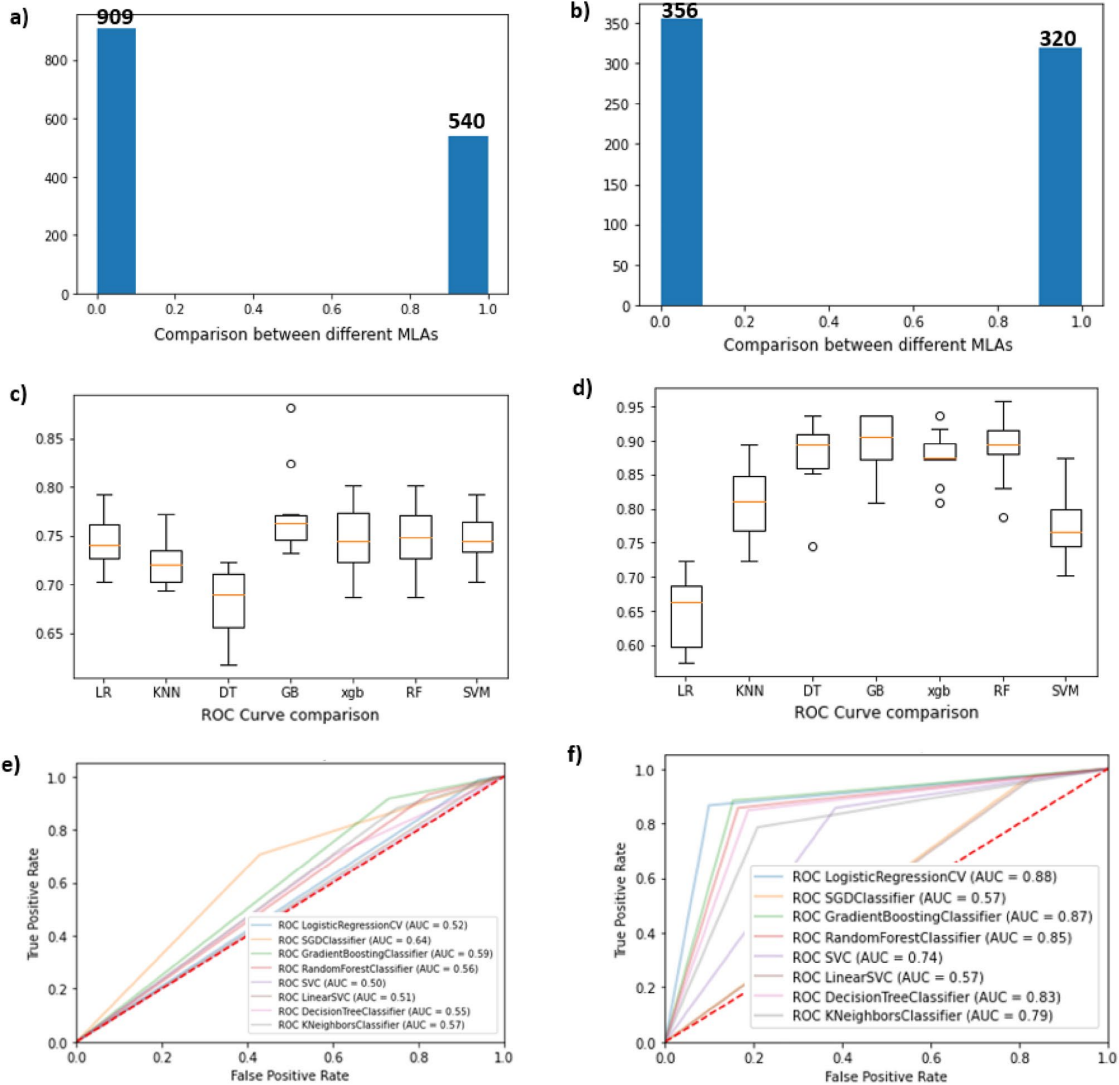
Comparative result analysis of various methods for the identification of the best machine learning models before and after the removal of outliers in the dataset (**a** and **b**) describes the number of clusters k representing the matching score of variables (**c** and **d**) A comparative analysis of various machine learning models and (**e** and **f**) Comparative analysis of ROC.

The blood profile data of cancer patients were obtained from the cancer hospital and the normal blood profile was collected from different hospitals as a part of regular health check-ups. A sample of 1073 cancer patients whose diagnosis had been confirmed with metastasis and 376 normal subjects. A total of 1449 data entries were tabulated as a single data frame. The variables considered were haemoglobin, red cell count, WBC count, neutrophils, lymphocyte, and monocyte counts. Total blood profile data without the categorical labels (Normal = 0, Cancer = 1) intersected with the medical record numbers (Supplementary Table 2) with the keywords of ‘metastasis’ and ‘lymph node’. Subsequently, matched records with supplementary Table 2 are labeled as ‘1’ and unmatched records are labeled as ‘0’. This labeled data is considered experimental data, which is subjected to a k-means clustering algorithm to predict the clusters. It has resulted a dataset of two different clusters with the predicted labels of ‘0’ and ‘1’, which were considered as a predicted dataset. As shown in Figs. 6a the strength of classes A and B were found to be 540 and 909 data points, respectively. Next, categorical labels of the experimental data is matched with the categorical labels of the predicted data. As shown in Fig. 6b the dataset is reduced to 676 entries (320 + 356) and this data set is considered to classify ‘Breast Cancer Metastasis’ using various machine learning models. Unmatched data points considered as outliers. To maintain the same unit of measure, the dataset was rounded using the round () function. For example, the reference haemoglobin range in normal subjects is 120.0–150.0. Hence, we processed the dataset using round (df ["Haemoglobin"]/150.0,2). Here, df is a data frame, 150.0 is the maximum haemoglobin content in normal subjects, and 2 refers to storing the data up to 2 decimal points. Before training datasets for machine learning models, a dimensionality reduction technique Principle Component Analysis (PCA) is employed to reduce the computational time. The train_test_split () method is used to split our data into train and test sets using scikit-learn's train_test_split (X,y,test_size = 0.3,random_state = 43). The X train dataset contains 473 instances and the X test dataset contains 203 instances and the y test value counts for label “1” was 112 and “0” was 91 instances, respectively. Our dataset is processed using nine distinct machine-learning models to select the best model. A five-fold cross-validation technique is applied to evaluate the performance of machine learning models (Fig. 6c and 6d). Accuracy, recall, precision, F1 score, and Area Under the Curve (AUC) for all the models are represented in (Fig. 6e and 6f) and Table 4.

**Table 4:** Performance of various machine learning algorithms using blood profile data for the classification of Breast cancer metastasis.

Classification models	Before removal of outliers					After removal of outliers				
	Accuracy	Recall	Precision	F1 Score	AUC	Accuracy	Recall	Precision	F1 Score	AUC
Logistic regression	0.73	0.98	0.73	0.84	0.74	0.61	0.96	0.59	0.73	0.68
KNN	0.7	0.87	0.75	0.81	0.73	0.78	0.78	0.81	0.79	0.79
Decision trees	0.64	0.75	0.75	0.75	0.68	0.83	0.86	0.83	0.85	0.87
Random forest	0.73	0.98	0.74	0.84	0.74	0.83	0.89	0.81	0.85	0.85
SVM linear	0.72	1	0.72	0.84	0.74	0.55	1	0.55	0.71	0.62
Polynomial SVM	0.73	0.99	0.73	0.84	0.74	0.82	0.86	0.82	0.84	0.85
Radial SVM	0.72	1	0.72	0.84	0.74	0.55	1	0.55	0.71	0.62
Gradient boosting	0.72	0.99	0.72	0.84	0.72	0.81	0.95	0.76	0.84	0.85
XGBOOST	0.73	0.99	0.73	0.84	0.7	0.78	0.93	0.74	0.83	0.85

The table represents the comparative performance of ML models before and after the removal of the outliers.

Based on the performances we conclude that Decision Tree (DT), Random Forest (RF), and Gradient Boosting (GB) models shown higher accuracy. Among all the DT classifier shown more than 83% accuracy with an AUC of 0.87, which is comparatively higher than other machine learning algorithms tested using our dataset.

### Web deployment and discussion

The decision tree model code with the highest accuracy was hosted on a remote server using a Flask framework. We used GoDaddy's (https://in.godaddy.com) server (Gen4 VPS Linux—1 CPU/1 GB RAM), putty, and WINSCP tools to create a web interface. The web interface is made up of three web pages. 1) The login page; 2) The page for entering a blood profile 3) The predictions page we added a limited email option to the login page with an administrator's permission to assure authenticity. The following HTML link can be used to access the web interface https://208.109.9.110:5000 for the prediction of cancer.

## Discussion

Extracting and analyzing the electronic health records (EHR) is a daunting task. Various text mining tools are available (https://monkeylearn.com/blog/text-mining-software/), to analyse the text documents. However, these tools cannot uncover the hidden relationships between the precise words of interest. For example, Clinicians at Basavatarakam Indo-American Cancer Hospital and Research Centre use Neura software, (https://cgslimited.com/clients/), to generate EHR of patients and The Epic Electronic Medical Record System is used at Johns Hopkins Medicine is used for clinical documentation. It suggest that different hospitals use different software`s to encrypt the index and clinical notes of their patients. Hence, health sector organizations approaching data analysts to develop in-house testing algorithms for a deep understanding of clinical data not only to closely monitor the patient's health but also monitor overall well-being of patients. In this paper, we developed an in-house text-mining framework to extract meaningful information and blood profile data using histopathological report (HR) of individual patients, which consist of the clinical, specimen, microscopic findings, impression, and gross findings. Clinical information is based on mammography and other imaging techniques like PET and MRI techniques, which result in the presence or absence of benign or malignant tumors in either the right or left breast^49^. Specimen details consist of a biopsy from the right or left breast, a chest wall lesion, and an axillary or sentinel lymph node. Microscopic impressions provide detailed information about the histological observations of the cancer cell. We used the word cloud visualization technique to analyze the most frequent words used in microscopic and overall gross findings. Microscopic and gross findings revealed that the most frequent words used by pathologists are hyperchromatic nuclei, nucleus to cytoplasm ratio, nuclear pleomorphic, mitotic count, invasiveness, and Richardson score. Each of these cellular features are clinically important biomarker in determining the prognosis of invasive breast cancer^50^. Based on our text mining and analytics, it is clear that patients with ductal carcinoma are more prevalent than any other type of cancer, with 49% of cases involving lymph nodes and metastasis. We used a web driver from Selenium to identify the association of blood parameters (monocytes, neutrophils, and lymphocytes) with cancer-specific characteristics ("nuclear-pleomorphism", "mitotic-nuclei", "hyperchromatic nuclei", "infiltrate", "eosinophilic", "vascular", "metastasis", "Richardson-score", "metastatic", "invasion", "ductal-carcinoma", "bone", "liver", and "brain") in the progression of breast cancer.

**Figure 7:**
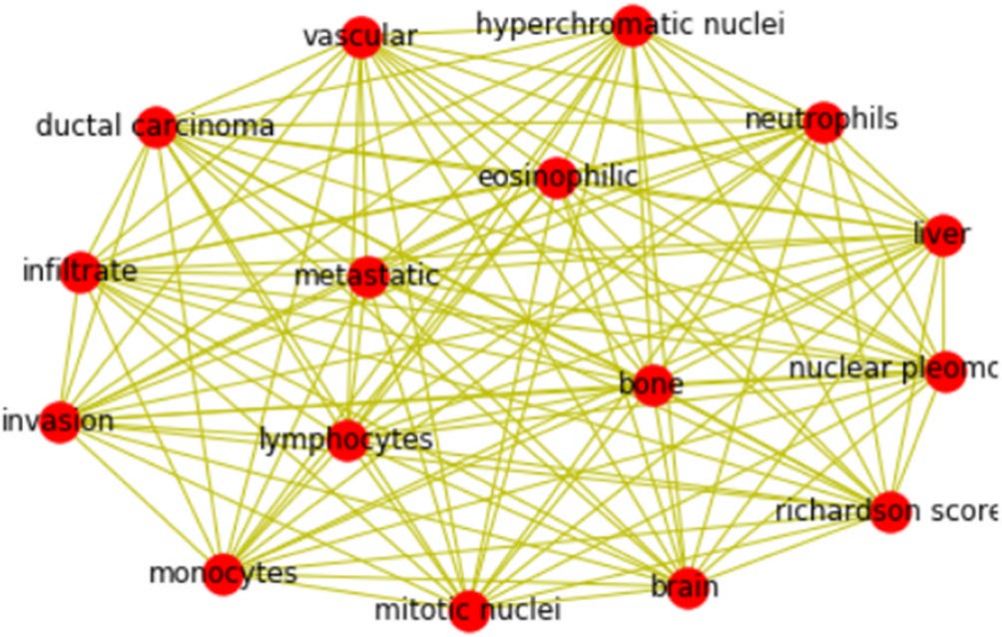
The Word Cloud Generator Based on Text Network Visualization. Closely located words represent terms frequently used together to classify cancer.

The association of words with cancer characters is plotted using in-house developed Python code as shown in Fig. 7. As shown in the figure, it is clear that blood parameters are indefinitely associated with cancer characteristics and their careful examination possibly helps in the easy diagnosis of breast cancer. The automatic text-mining functionality of VOSviewer and Word2vec is used for the classification of decision making variable in breast cancer surgery and to retrieve co-occurrence networks of terms associated with PCA^51,52^.

Based on text mining, we retrieved medical record numbers with “metastasis” and “lymph node” involvement. Similar approaches were used to identify the hub genes for cancer metastasis^53^. Dissemination of cancer cells from the primary site via the blood is called metastasis^54,55^. The metastasis of a tumor is always accompanied by inflammation, and haematological inflammatory markers have been used to diagnose the advanced stage and grade of various tumors^56^. To develop a blood-based breast cancer detection method, blood profile data from histologically confirmed metastasis patients was used. We found a clear distinguishing pattern by analyzing an equal number of patients as compared to normal. The mean difference for monocyte count in cancer patients (7.76) is higher than the normal range (2–6%). This may be due to the presence of circulatory monocytes in high-grade breast cancer patients^57^. On the other hand, we found a mean differential count in total WBC and lymphocyte count. It may be due to systemic inflammation and immune suppression in metastatic breast cancer patients. Our data is in accordance with a recently published article demonstrating Neutrophil to Lymphocyte Ratio (NLR) and Monocyte To Lymphocyte Ratio (MLR) are used biomarkers for predicting metastatic breast cancer^58^. To find out the influence of chemotherapy on these cells, we retrospectively collected blood profiles from 40 chemotherapy patients. Cycle-wise means the difference is calculated, as shown in Fig. 3 monocyte number increased until the 3^rd^ cycle of AC and gradually reached the baseline. A decrease in peripheral blood monocytes during or after chemotherapy is a possible predictor of neutropenia^59^. It suggests that AC chemotherapy did not cause neutropenia. Overall, it suggests that cancer patients display a distinguishing pattern of peripheral blood components, and this pattern can be used for the classification of advanced breast cancer using machine learning techniques^60^. Breast cancer metastasis was predicted using serum biomarkers and clinicopathological data with machine learning technologies^61^.

One of the most effective ways to build a Clinical Decision Support System (CDSS) is to collect and analyze large amounts of evidence-based clinical research findings in the appropriate context. CDSS helps health professionals to make accurate recommendations, which improves the survival outcome of patients with advanced breast cancer^62^. Machine learning techniques help to build a clinical decision-support system that helps health professionals make clinical decisions. One such tool built by entrepreneurs is Niramai software. Niramai is a portable cancer screening tool that is used to diagnose breast cancer and is based on thermal image processing (https://www.niramai.com/). Machine learning algorithms also used to identify predictive molecular markers for cisplatin chemosensitivity and plasma lipid markers for ovaraian and gastric cancer respectively^63,64^. In this paper, for the first time, we predicted breast cancer using blood profile counts and clinicopathological data. As a first step, blood parameters from 376 normal and 1073 cancer subjects were collected and prepared as a single dataset. Various modules such as NumPy, Pandas, Sklearn, Matplotlib, and SciPy are used for visualizing the data and understanding the correlation between each feature. It is known from the comparative cycle-wise mean value distribution for haematological parameters is that blood profile data is responsive to chemotherapy regime. We do not know whether patient is undergoing any chemotherapy regime at the time of data collection. Hence, we employed K means unsupervised clustering technique to remove the outliers Next, we fit our dataset into various machine-learning algorithms for the prediction of breast cancer metastasis using blood profile data with and without outliers. Initially, we fitted our dataset into the LR model to predict the model performance. Classification accuracy was found to be 0.61. Low accuracy may be due to relationships between multiple variables and attributes in our dataset, and LR could not handle the dataset with the correlated features^66^. Next, we employed our data set in the K-mean clustering algorithm using the Minkowski distance metric with the number of neighbors as 5. The accuracy is 0.78, which is greater than LR. Although KNN is widely used for binary classification models for the prediction of cancer^65^, with our dataset we could not achieve accuracy greater than or equal to 80%. It may be due to a small data set. In a clinical setting, small data is acceptable because it is difficult to get clinical information from cancer patients with low blood volume and haemoglobin content^66^. SVM models are a good fit for small-to-intermediate datasets with a manageable number of features. Hence, we fitted our dataset into SVM and compared the results using various kernels (linear, polynomial, and radial). Results showed that the accuracy of SVM linear is 0.55, the polynomial is 0.82, and the radial is 0.55. So we decided to apply our dataset to other ensemble learning algorithms. We found that the accuracy of the decision tree (0.83), random forest (0.83), GB (0.81), and XGB are, respectively. Overall, it suggests that decision trees, random forest, and SVM with kernel polynomial machine learning models established relationships between cancer and normal subjects with a high degree of accuracy. Among all, the ensemble-based decision tree method is further deployed for web deployment.

## Conclusion

Metastatic breast cancer is the major cause of cancer death in women. It is mainly due to a lack of early diagnosis. Based on our extensive data analysis we conclude that blood profile data can be used as a non-invasive machine learning method for early diagnosis of breast cancer metastasis. Among nine algorithms tested the Decision Tree (DT) classifier displayed an accuracy of 83% as compared to the ensemble and logistic regression models. Although, the obtained accuracy rate cannot be regarded as very high, it can be improved by increasing the number of attributes, which include liver and kidney function tests, serum biomarkers, vitamin D3 and Vitamin B12 levels. Our future work is related to implementing our web interface across the multi-speciality hospitals not only to validate our hypothesis but also to generate a cancer-specific database with more attributes. We can develop a precision medicine platform with a single drop of blood by deploying various statistical methods and machine learning models to increase the overall survival rate and decrease healthcare expenditures for cancer patients.

## Supplementary Information

Supplementary Table S1.

Supplementary Table S2.

Supplementary Table S3.

Supplementary Table S4.

Supplementary Table S5.

## Data Availability

This article and its supplementary information files contain entire data generated or analyzed during the course of this investigation and are made available to the readers.
